# Associations of Body Fat Distribution and Cardiometabolic Risk of Testicular Cancer Survivors After Cisplatin-Based Chemotherapy

**DOI:** 10.1093/jncics/pkac030

**Published:** 2022-05-13

**Authors:** Andreas G Wibmer, Paul C Dinh, Lois B Travis, Carol Chen, Maria Bromberg, Junting Zheng, Marinela Capanu, Howard D Sesso, Darren R Feldman, Hebert Alberto Vargas

**Affiliations:** Department of Radiology, Memorial Sloan Kettering Cancer Center, New York, NY, USA; Department of Medicine, Indiana University School of Medicine, Indianapolis, IN, USA; Department of Medicine, Indiana University School of Medicine, Indianapolis, IN, USA; Department of Epidemiology, Fairbanks School of Public Health, Indiana University, Indianapolis, IN, USA; Department of Medicine, Cardiology Service, Memorial Sloan Kettering Cancer Center, New York, NY, USA; Department of Medicine, Genitourinary Service, Memorial Sloan Kettering Cancer Center, New York, NY, USA; Department of Epidemiology and Biostatistics, Memorial Sloan Kettering Cancer Center, New York, NY, USA; Department of Epidemiology and Biostatistics, Memorial Sloan Kettering Cancer Center, New York, NY, USA; Department of Medicine, Brigham and Women’s Hospital, Boston, MA, USA; Department of Medicine, Genitourinary Service, Memorial Sloan Kettering Cancer Center, New York, NY, USA; Department of Radiology, Memorial Sloan Kettering Cancer Center, New York, NY, USA

## Abstract

**Background:**

It is unknown how body fat distribution modulates the cardiometabolic risk of testicular cancer survivors after cisplatin-based chemotherapy.

**Methods:**

For 455 patients enrolled in the Platinum Study at Memorial Sloan Kettering Cancer Center, visceral (VAT) and subcutaneous (SAT) adipose tissue was quantified on prechemotherapy computed tomography. The VAT-to-SAT ratio was calculated as a quantitative measure of central adiposity. Endpoints were incidence of new posthemotherapy cardiometabolic disease (new antihypertensive, lipid-lowering, or diabetes medication), and postchemotherapy Framingham risk scores. Cox models and linear regression with interaction terms were applied. Postchemotherapy body fat distribution was analyzed in 108 patients. All statistical tests were 2-sided.

**Results:**

The baseline median age was 31 years (interquartile range [IQR] = 26-39 years), body mass index (BMI) was 26 kg/m^2^ (IQR = 24-29 kg/m^2^), and the VAT-to-SAT ratio was 0.49 (IQR = 0.31-0.75). The median follow-up was 26 months (IQR = 16-59 months). Higher prechemotherapy VAT-to-SAT ratios inferred a higher likelihood of new cardiometabolic disease among patients with a BMI of 30 kg/m^2^ or greater (age-adjusted hazard ratio = 3.14, 95% confidence interval = 1.02 to 9.71, *P* = .047), but not other BMI groups. The prechemotherapy VAT-to-SAT ratio was associated with postchemotherapy Framingham risk scores in univariate regression analysis (exp(β)-estimate: 2.10, 95% confidence interval = 1.84 to 2.39, *P* < .001); in a multivariable model, this association was stronger in younger vs older individuals. BMI increased in most patients after chemotherapy and correlated with increases in the VAT-to-SAT ratio (Spearman *r* = 0.39, *P* < .001).

**Conclusions:**

In testicular cancer survivors, central adiposity is associated with increased cardiometabolic risk after cisplatin-based chemotherapy, particularly in obese or young men. Weight gain after chemotherapy occurs preferentially in the visceral compartment, providing insight into the pathogenesis of cardiovascular disease in this population.

Most advanced male germ cell tumors respond to platinum-based chemotherapy, and nearly 80% of patients achieve long-term survival (1). Given their generally young age at diagnosis, testicular or extragonadal germ cell tumor survivors, hereafter referred to as testicular cancer survivors (TCSs), enjoy a long life expectancy; hence, long-term adverse health outcomes related to cancer therapy are of special concern (2). Although the exact pathophysiologic mechanisms are not well understood, it is recognized that platinum-based chemotherapy puts TCSs at a considerably increased risk for cardiometabolic disease (CMD) (3-5). However, the risk is not equally distributed across all patients, and identifying individuals who might benefit from targeted intervention and risk modulation is challenging because of the low prevalence of established risk factors (eg, hypertension, dyslipidemia, diabetes) before chemotherapy in this young population. Although it is widely accepted that obesity is associated with increased CMD risk in the general population (6), recent research emphasizes the relative importance of body fat distribution over total fat mass (7,8). A recurrent finding in these studies is that preferential fat accumulation in the intra-abdominal visceral compartment relative to the subcutaneous compartment (ie, “central adiposity”) is associated with increased CMD risk (7). This relationship was recently summarized in a meta-analysis encompassing prospective data from 2.5 million individuals showing that central adiposity, independent of overall adiposity, is closely associated with all-cause mortality in the general population (8). Central adiposity has also been identified as a risk factor for CMD after cancer treatment in survivors of breast cancer (9,10) and childhood brain cancer (11). For TCSs, Hashibe et al. (12) found an association of overall adiposity (estimated from driver’s license records) and CMD risk, but this has not been validated by others, and the extent to which adipose tissue distribution affects cardiometabolic health of TCSs is not known.

To address these gaps, we quantified VAT and SAT compartments of TCSs on prechemotherapy computed tomography (CT) scans and tested whether quantitative metrics of fat distribution and central adiposity could be used to more accurately estimate their cardiometabolic risk after chemotherapy.

## Methods

### Study Cohort

**Figure 1.**
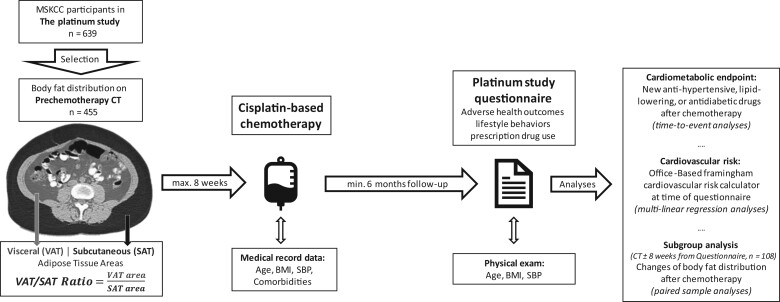
Study flow chart. BMI = body mass index; CT = computed tomography; max. = maximum; min. = minimum; MSKCC = Memorial Sloan Kettering Cancer Center; SAT = subcutaneous adipose tissue, SBP = systolic blood pressure; VAT = visceral adipose tissue.

All 639 TCSs who were enrolled on the Platinum Study at Memorial Sloan Kettering Cancer Center (MSKCC) were screened for eligibility. The Platinum Study is a multicenter prospective clinical investigation of long-term morbidities among cisplatin-treated TCSs in 8 centers in the United States, Canada, and the United Kingdom (13). Platinum Study eligibility criteria were previously described (14,15), but briefly included age 18 years and older, cancer diagnosis before age 55 years, male gender, first-line cisplatin-based chemotherapy completed at least 6 months before enrollment, no subsequent salvage chemotherapy, and no radiation or previous chemotherapy. Of the 639 patients, 455 (71.2%) had an abdominal CT within 8 weeks before chemotherapy initiation available at MSKCC and were eligible for further study (see Figure 1). The MSKCC Internal Review Board approved the study, and all patients provided written informed consent for medical record access.

### Data Collection

All participants completed a questionnaire at least 6 months postchemotherapy that included adverse health outcomes, lifestyle behaviors, and prescription medication use, as previously detailed (13). At this time, participants also underwent a physical exam, including height, weight, and systolic blood pressure (SBP). For the current analysis, clinical data at chemotherapy initiation (ie, regimen, weight, SBP, comorbidities, prescription drugs) were extracted from the medical record and/or from the Platinum Study questionnaire. CT scans were analyzed with commercially available software (Aquarius iNtuition version 4.4.13.; Tera Recon, Foster City, CA, USA). Visceral (VAT) and subcutaneous (SAT) adipose tissue areas (CT density range: −195 to −45 Hounsfield units) were semiautomatically extracted in the transverse plane at the L3/4 intervertebral level. This methodology was based on evidence from the Framingham Heart Study that 2-dimensional measurements at L3/4 most strongly correlate with abdominal fat volumes and cardiometabolic risk (16). The VAT-to-SAT ratio was calculated as a quantitative measure of central adiposity, with higher ratios indicating greater central adiposity. As a reference, the median VAT-to-SAT ratio of male patients (mean age ± SD = 49.5 ± 10.6 years) in an analysis from the Framingham Heart Study was reported to be 0.84 (interquartile range [IQR] = 0.64-1.10) (17).

### Endpoints

The primary study endpoint was new-onset CMD after chemotherapy completion, defined as emergence of any of the following between the end of chemotherapy and Platinum Study questionnaire completion: 1) arterial hypertension, 2) dyslipidemia, and 3) type 2 diabetes (ie, composite cardiometabolic endpoint). Diagnosis of each comorbidity required self-reporting of new prescription medication use for treatment. Patients taking medications for CMD prechemotherapy were excluded from this part of the analysis. The secondary endpoint was the association between baseline variables and the office-based Framingham risk score (FRS) as assessed at the time of questionnaire completion (18). Changes in body fat distribution after chemotherapy were analyzed in 108 patients who underwent a CT scan within ±8 weeks of questionnaire completion.

### Statistical Methods

We analyzed 2 outcomes, time to new-onset CMD and postchemotherapy FRS, using the Cox proportional hazard regressions and linear regressions, respectively. Associations between new-onset CMD and baseline variables (prechemotherapy age, SBP, body mass index [BMI], logarithmic transformed VAT-to-SAT ratio, existing CMD) were examined using univariate Cox models. With a small event number, multivariable analyses were used to examine a base model with BMI group (ie, <25, 25-29, ≥30 kg/m^2^), VAT-to-SAT ratio, and their interaction term as well as a model adding prechemotherapy age to account for its confounding effect. Complete data were used considering the rarity of missing data (0.4% and 2.0% patients missing prechemotherapy BMI and SBP, respectively). The proportional hazards assumption was satisfied in all Cox models based on time-weighted score tests (19). Univariate and multivariable linear regressions were used to examine associations between postchemotherapy FRS (logarithmic transformed) with baseline variables and the time interval from chemotherapy to Platinum Study questionnaire. Exponentiated beta coefficients from linear models (exp(ß)-estimate to account for the log-transformation of FRS) were used to report the degree of fold change in FRS for every 1 unit of change (or fold change) in a predictor variable (20,21). The multivariable model included variables and 2-way interaction terms selected by backward selection with *P* values less than .05.

Comparison of variables between 2 timepoints was performed with paired *t* test or the Wilcoxon signed rank test. The Spearman correlation coefficient was used to assess correlations between continuous variables, and the χ^2^ test or the Wilcoxon rank-sum test was used to test variable differences between patients with or without follow-up CT. Statistical analyses were performed in software packages SAS 9.4 (SAS Institute Inc., Cary, NC, USA) and R 4.0 (The R Foundation for Statistical Computing). All statistical tests were 2-sided, and a *P* less than .05 was considered statistically significant.

## Results

### Cohort Characteristics

Cohort characteristics are shown in Table 1. At chemotherapy initiation, median age was 31 years (IQR = 26-39 years) and median BMI was 26 kg/m^2^ (IQR = 24-29 kg/m^2^). Age and BMI at chemotherapy start were positively correlated (*r* = 0.20, *P* < .001). Thirty-eight patients (8.4%) had been diagnosed with and treated for CMD before chemotherapy start, including 16 patients (3.5%) on antihypertensive medications, 27 (5.9%) on lipid-lowering drugs, and 3 (0.66%) on antidiabetic medication. All patients received cisplatin-based chemotherapy in combination with etoposide (n = 358, 78.6%), bleomycin and etoposide (n = 41, 9.0%), paclitaxel and ifosfamide (n = 34, 7.5%), or in other combinations (n = 22, 4.8%).

**Table 1.** Characteristics of 455 GCT survivors for whom visceral and subcutaneous fat was quantified on pre-chemotherapy CT scans

Characteristics	Values
At time of chemotherapy initiation	
Median age (IQR; range), y	31 (26-39; 15-58)
Race, No. (%)	
Asian	16 (3.5)
Black or African American	7 (1.5)
Hispanic or Latino	33 (7.3)
Other^a^	4 (0.9)
White	381 (83.7)
Unspecified	14 (3.1)
Median BMI (IQR; range), kg/m^2^	26 (24-29; 16-49)
BMI group, No. (%)	
Not available	2 (0.44)
<25 kg/m^2^	173 (38)
≥25 to <30 kg/m^2^	178 (39)
≥30 kg/m^2^	102 (22)
Median systolic blood pressure(IQR; range), mmHg	122 (114-130; 91-168)
Cardiometabolic disease, No. (%)	38 (8.4)
Antihypertensive medication	16 (3.5)
Lipid-lowering medication	27 (5.9)
Type 2 diabetes medication	3 (0.66)
Chemotherapy regimen, No. (%)	
EP	358 (79)
6 cycles	2 (0.44)
4 cycles	330 (73)
2 cycles	26 (5.7)
BEP	41 (9.0)
4 cycles	26 (5.7)
3 cycles	14 (3.1)
2 cycles	1 (0.22)
TIP	
4 cycles	34 (7.5)
Other regimens^b^	22 (4.8)
CT and body fat distribution, median (IQR; range)	
Time from CT tochemotherapy, d	13 (6-21; 0-56)
SAT area^c^, cm^2^	156 (100-228; 1.8-760)
VAT area^c^, cm^2^	75 (37-140; 2.5-490)
VAT-to-SAT ratio	0.49 (0.31-0.75; 0.06-5.2)
At completion of Platinum Study Questionnaire^d^	
Chemotherapy to questionnaireinterval, median (IQR;range), mo	26 (16-59; 7.1-207)
Median age (IQR; range), y	35 (29-43; 18-66)
Median BMI (IQR; range), kg/m^2^	27 (25-31; 18-54)
BMI group, No. (%)	
<25 kg/m^2^	126 (28)
≥25 to <30 kg/m^2^	204 (45)
≥30 kg/m^2^	125 (27)
Median systolic blood pressure(IQR; range), mmHg	121 (114-129; 94-171)
Cardiometabolic disease, No. (%)	59 (13)
Antihypertensivemedication	30 (6.6)
Lipid-lowering medication	34 (7.5)
Type 2 diabetes medication	10 (2.2)
Median Framingham Risk^e^ (IQR;range), %	3.3 (1.7-7.0; 0.27-38)

a Includes Native American, Pacific Islander, and unspecified (patient declined to report).

b BEP×3+EP×1 (n = 5); BEP×2+EP×2 (n = 3); EP×2+EC2, EP×3+EC×1, TIP×1+BEP×3 (n = 2 each); BEP×1+EP×2, BEP×1+EP×3, BEP×2+EP×1, EC×2+BEP×1+TIP×3, EP×1+VIP×3, EP×1+VIP×4, EP×4+TIP×2, TIC×1+TIP×3 (n = 1 each).

c Measured in transverse plane at level of intervertebral space L3/4.

d Platinum Study questionnaire and concurrent physical examination.

e Framingham Heart Study estimated 10-year risk for atherosclerotic cardiovascular disease (office-based calculator).

BEP = bleomycin, etoposide, cisplatin; BMI = body mass index; CT = computed tomography; EC = etoposide, cyclophosphamide; EP = etoposide, cisplatin; GCT = germ cell tumor; IQR = interquartile range; SAT = subcutaneous adipose tissue; TIC = paclitaxel, ifosfamide, carboplatin; VAT = visceral adipose tissue; VIP = etoposide, ifosfamide, cisplatin.

### Fat Quantification on Prechemotherapy CT

The median time from CT to chemotherapy initiation was 13 days (IQR = 6-21 days). On CT, the median VAT and SAT areas were 75 cm^2^ (IQR = 37-140 cm^2^) and 156 cm^2^ (IQR = 100-228 cm^2^), respectively, and the median VAT-to-SAT ratio was 0.49 (IQR = 0.31-0.75). Whereas strong correlations were found between VAT and SAT areas (Spearman *r* = 0.73, *P* < .001), between BMI and VAT (Spearman *r* = 0.65, *P* < .001), and between BMI and SAT (Spearman *r* = 0.77, *P* < .001), the correlation between the VAT-to-SAT ratio and BMI was weak (*r* = 0.09, *P* = .050). Higher VAT-to-SAT ratios correlated with older age (Spearman *r* = 0.48, *P* < .001) and higher SBP (Spearman *r* = 0.13, *P* = .004) and were associated with preexisting CMD (odds ratio = 1.96, 95% confidence interval [CI] = 1.11 to 3.48, *P* = .02).

### New-Onset CMD After Chemotherapy

The median time between chemotherapy and questionnaire completion was 26 months (IQR = 16-59 months). The estimated incidence of new postchemotherapy CMD was 3.7% (95% CI = 2.1% to 5.7%) at 2 years and 8.2% (95% CI = 5.0% to 12%) at 5 years. In univariate analyses, baseline age (hazard ratio [HR] per year = 1.08, 95% CI = 1.05 to 1.12, *P* < .001), BMI (HR per kg/m^2^ = 1.11, 95% CI = 1.05 to 1.17, *P* = .001), and SBP (HR per 10 mmHg = 1.52, 95% CI = 1.18 to 1.96, *P* = .003) were associated with a higher likelihood of new-onset CMD.

**Figure 2.**
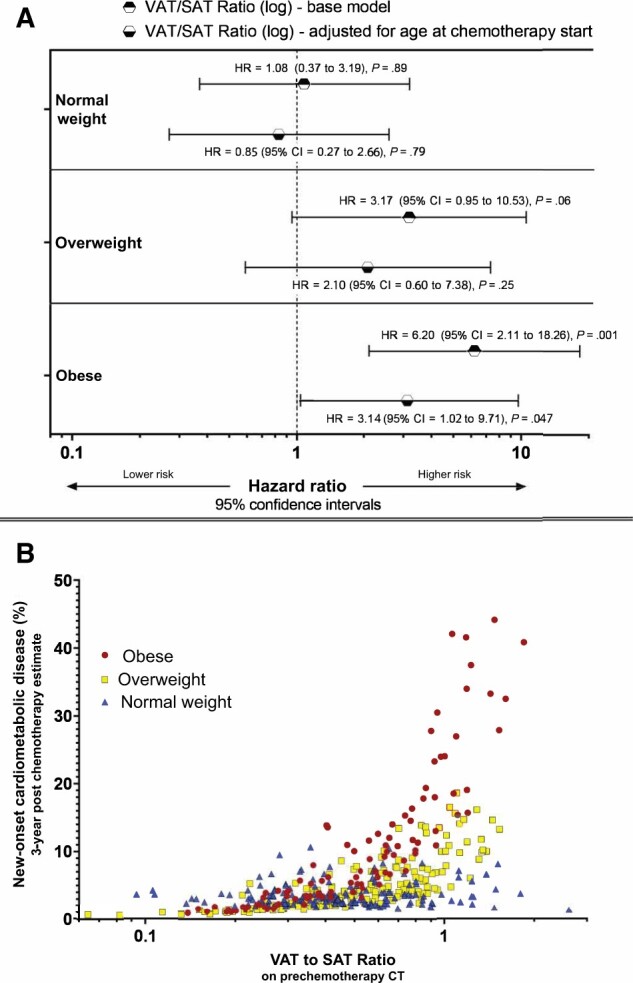
Associations between body fat distribution and new-onset cardiometabolic disease (CMD) after cisplatin-based chemotherapy. **A**) Associations between visceral adipose tissue (VAT) to subcutaneous adipose tissue (SAT) ratio on prechemotherapy computed tomography (CT) and the risk of new-onset CMD after cisplatin-based chemotherapy in normal or underweight (ie, body mass index [BMI] < 25 kg/m^2^), overweight (BMI ≥ 25-29 kg/m^2^), and obese (BMI ≥ 30 kg/m^2^) testicular cancer survivors (TCSs). Results from base model (**blue**) and age-adjusted model (**red**). Hazard ratios (HRs) are given with 95% confidence intervals (CIs) as error bars (given as data within parentheses). *P* values are based on the 2-sided Wald test. **B**) Estimated probability of new-onset CMD (new antihypertensive, lipid-lowering, or antidiabetic medication) in normal or underweight, overweight, and obese TCSs at 3 years after cisplatin-based chemotherapy as a function of the VAT-to-SAT ratio on prechemotherapy CT. The estimates are derived from the age-adjusted statistical model detailed in Table 2.

Figure 2, A and Table 2 illustrate associations between the prechemotherapy VAT-to-SAT ratio and the composite CMD endpoint separately for the 3 BMI groups. In obese individuals, a higher VAT-to-SAT ratio was consistently associated with a greater likelihood of new-onset CMD both in the base model (HR = 6.20, 95% CI = 2.11 to 18.26, *P* = .001) and age-adjusted model (HR = 3.14, 95% CI = 1.02 to 9.71, *P* = .047). In overweight patients, there was a trend toward higher CMD risk with increasing VAT-to-SAT ratio on univariate analysis (HR = 3.17, 95% CI = 0.95 to 10.53, *P* = .06), but this effect was reduced after adding prechemotherapy age as a covariate (HR = 2.10, 95% CI = 0.60 to 7.38, *P* = .25). For men with BMI < 25 kg/m^2^, there was no association between the VAT-to-SAT ratio and postchemotherapy CMD. Figure 2, B displays the estimated risk of new CMD at 3 years after chemotherapy start as a function of the VAT-to-SAT ratio at baseline.

**Table 2.** Associations between the prechemotherapy VAT-to-SAT ratio^a^ and new-onset cardiometabolic disease after chemotherapy^b^

BMI category	Base model		Age-adjusted model	
	Hazard ratio (95% CI)	*P* ^c^	Hazard ratio (95% CI)	*P* ^c^
Normal weight (<25 kg/m^2^)	1.08 (0.37 to 3.19)	.89	0.85 (0.27 to 2.66)	.79
Overweight (25-29 kg/m^2^)	3.17 (0.95 to 10.53)	.06	2.10 (0.60 to 7.38)	.25
Obese (≥30 kg/m^2^)	6.20 (2.11 to 18.26)	.001	3.14 (1.02 to 9.71)	.047

a The VAT to SAT ratio is measured on prechemotherapy computed tomography. BMI = body mass index; CI = confidence interval; SAT = subcutaneous adipose tissue; VAT = visceral adipose tissue.

b Results of base and age-adjusted Cox regression models. Graphical representation in Figure 2, A.

c *P* values are based on the 2-sided Wald test.

### Framingham-Estimated Cardiovascular Risk After Chemotherapy

**Figure 3.**
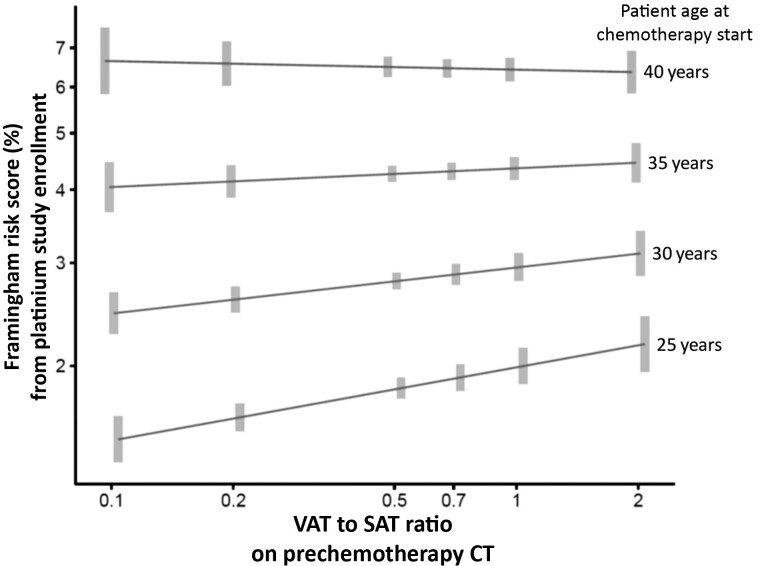
Interaction of prechemotherapy visceral adipose tissue (VAT) to subcutaneous adipose tissue (SAT) ratio and age in their association with postchemotherapy Framingham risk score at the time of the Platinum Study questionnaire. A higher VAT-to-SAT ratio had a stronger effect in younger vs older patients.

At the time of questionnaire completion postchemotherapy, the median FRS was 3.3% (IQR = 1.7% to 7.0%) and statistically significantly associated with baseline age, BMI, SBP, the time interval between chemotherapy and questionnaire (*P* < .001 for all, Table 3), and the VAT-to-SAT ratio on prechemotherapy CT (exp(β)-estimate = 2.10, 95% CI = 1.84 to 2.39, *P* < .001; Table 3). In a multivariable model including statistically significant interaction terms in addition to these covariates, the detrimental effect of a higher VAT-to-SAT ratio on postchemotherapy FRS was stronger in younger vs older individuals (Table 3; Figure 3).

**Table 3.** Associations between prechemotherapy characteristics and interaction terms with postchemotherapy Framingham risk scores^a^

Variable	Framingham-estimated risk of cardiovascular disease within 10 y after study questionnaire^a^			
	Univariate analyses		Multivariable analysis	
	exp(β)-estimate (95% CI)	*P* ^c^	exp(β)-estimate (95% CI)	*P* ^c^
Age^b^ (per 5-y increase)	1.57 (1.54 to 1.61)	<.001	1.53 (1.48 to 1.57)	<.001
Time interval between chemotherapy and questionnaire (per 1-y increase)	1.09 (1.07 to 1.12)	<.001	1.17 (1.13 to 1.20)	<.001
BMI^c^ (per 1-kg/m^2^ increase)	1.07 (1.06 to 1.09)	<.001	1.04 (1.04 to 1.05)	<.001
Systolic blood pressure^c^ (per 10-mmHg increase)	1.26 (1.17 to 1.35)	<.001	1.07 (1.05 to 1.10)	<.001
VAT-to-SAT ratio^d^ (per e [2.72] fold increase)	2.10 (1.84 to 2.39)	<.001	1.43 (1.21 to 1.69)	<.001
Interaction: age × time interval	–	–	0.99 (0.987 to 0.995)	<.001
Interaction: age × VAT-to-SAT ratio	–	–	0.95 (0.93 to 0.98)	<.001

a Framingham Heart Study estimated 10-year risk for atherosclerotic cardiovascular disease (office-based calculator) (logarithmic transformed). BMI = body mass index; CI = confidence interval; SAT = subcutaneous adipose tissue; VAT = visceral adipose tissue.

b *P* values are based on the 2-sided Wald test.

c At initiation of chemotherapy.

d On prechemotherapy computed tomography.

### Subgroup Analysis: Changes in Body Fat Distribution After Chemotherapy

**Figure 4.**
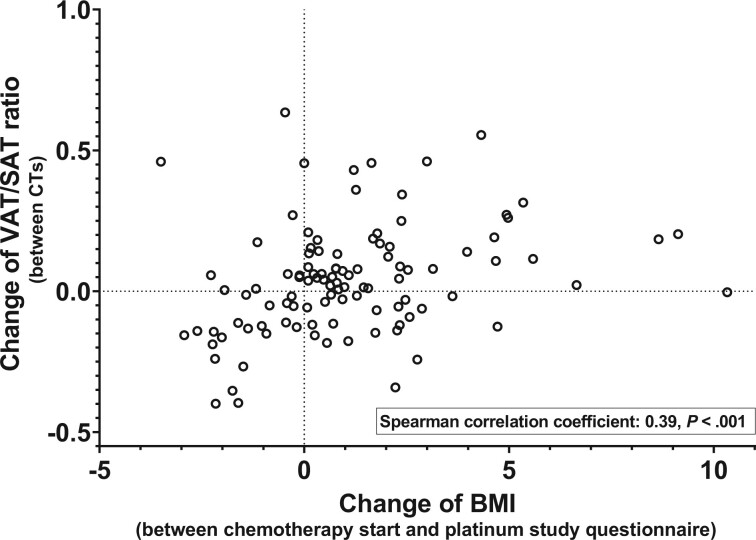
Association between changes in body mass index (BMI) and changes of the visceral adipose tissue (VAT) to subcutaneous adipose tissue (SAT) ratio in testicular cancer survivors (TCSs) after cisplatin-based chemotherapy (Spearman correlation coefficient = 0.39, *P* < .001). *P* values are based on the 2-sided Wald test.

Of the 455 patients, 108 (23.7%) underwent a CT scan within ±8 weeks of questionnaire completion. Although the time between chemotherapy and questionnaire completion was shorter in these 108 patients vs those without an eligible follow-up CT (median 18 vs 36 months; *P* < .001), there were no other statistically significant differences in terms of clinical characteristics and fat distribution (Supplementary Table 1, available online). Despite the shorter median follow-up time, there was a statistically significant increase in BMI (median = +0.71 kg/m^2^, IQR = −0.40 to +2.30 kg/m^2^, *P* < .001) from before chemotherapy and time of questionnaire completion postchemotherapy. Concurrently, VAT (median = +16 cm^2^, IQR = −5.3 to +48 cm^2^, *P* < .001) and SAT (median = +27 cm^2^, IQR = +3.8 to +62 cm^2^, *P* < .001) areas increased statistically significantly, but there were no statistically significant changes of VAT-to-SAT ratios (median = +0.02, IQR = −0.12 to +0.14, *P* = .15). However, changes in BMI correlated positively with changes in the VAT-to-SAT ratio (*r* = 0.39, *P* < .001), indicating that weight gain preferentially occurred in the VAT compartment, whereas weight loss was commonly accompanied by favorable changes in body fat distribution (Figure 4). A higher VAT-to-SAT ratio on the follow-up CT correlated with a higher FRS at questionnaire completion (*r* = 0.59, *P* < .001).

## Discussion

To our knowledge, we demonstrate for the first time that preferential fat storage in the abdominal visceral compartment over the subcutaneous compartment (ie, “central adiposity”) at the time of chemotherapy initiation in TCSs is associated with a statistically significantly higher risk of subsequent CMD. In a subgroup analysis, we observed that the frequent weight gain observed after chemotherapy in TCSs tends to be accompanied by an unfavorable expansion of the visceral over the subcutaneous fat compartment. In contrast, individuals who lost weight after chemotherapy generally experienced an improvement in their body fat distribution. This study also shows for the first time, to our knowledge, that body fat distribution seems to be as important a cardiometabolic risk factor as overall body mass in TCSs, particularly in those who are obese. This implies that quantification of visceral and subcutaneous fat compartments before chemotherapy might help clinicians to more accurately identify patients in need for intensified intervention, for example, education, monitoring, and aggressive risk modulation. Importantly, because baseline CT scans are routinely performed before chemotherapy initiation, data on body fat distribution are readily accessible without additional radiation exposure or cost. Quantification of fat on CT can be performed expeditiously with built-in or open-source image-viewing software (22).

The identification of visceral adiposity as a cardiometabolic risk factor in cisplatin-treated TCSs corroborates research in the general population (7,8) and in survivors of other cancer types (9–11). In a prospective analysis of more than 27 000 male and female participants in the INTERHEART study, Yusuf et al. (7) found that independent of overall body mass, central adiposity was a statistically significant risk factor for myocardial infarction. These associations were corroborated in a recent meta-analysis investigating the effects of body fat distribution on overall mortality in the general population (8) and emphasize the importance of body fat distribution in addition to overall body mass when assessing an individual’s cardiometabolic risk.

In contrast to studies in the general population reporting a statistically significant association between central adiposity and cardiovascular disease across BMI ranges (7,23), our results indicate the effect of central adiposity was more pronounced in obese male TCSs. The younger age distribution of our cohort (median age = 31 years) relative to these other series (median age = 60-66 years) (7,23) may provide 1 explanation for these differences, given that the relative influence of fat depots on cardiometabolic risk may differ across age groups (24,25). Ali and colleagues (24), for example, reported that in children and adolescents, unlike in adults, subcutaneous adiposity was more strongly and positively associated with cardiometabolic risk than visceral fat. Similarly, Kjellberg et al. (25) observed a stronger association of subcutaneous fat with metabolic risk factors in children. In support of this hypothesis, we observed a statistically significant correlation of prechemotherapy age and BMI in our cohort of TCSs. However, we also observed that central adiposity holds greater prognostic importance in younger vs older TCSs. This again is in line with studies in the general population that show the associations of mortality with overall adiposity (26,27) and central adiposity (8) weaken with advancing age. Finally, chemotherapy may induce CMD through a different mechanism and pathophysiology than in the general population and could modulate interactions between age, BMI, and body fat distribution. Chemotherapy has also been proposed to accelerate the aging process, which could exert a greater effect on younger rather than older adults (28). Finally, older patients likely develop CMD in a shorter timeframe, and the relatively short follow-up period of this study might have led to an overestimation of their CMD risk compared with younger individuals.

Many TCSs gain weight after cisplatin-based chemotherapy, as shown previously in both the Platinum Study (13) and the Norwegian cohort (29). The relative changes of VAT and SAT compartments in TCSs, however, are less well studied. One small investigation of 19 patients found a statistically significant increase in both visceral and subcutaneous fat compartments on magnetic resonance imaging 9 months after cisplatin-based chemotherapy (30), but longer-term studies were not conducted. Our observation that weight gain after chemotherapy tends to be accompanied by increased central adiposity in TCSs resembles previous observations of tape-measured waist-to-hip ratio changes in young men from the general population (31). Therefore, the preferential increase in visceral fat is probably only one of many factors in the pathogenesis of CMD in TCSs after cisplatin-based chemotherapy (3,32–34).

To our knowledge, this is the only study to date to quantify CT-determined body fat distribution in TCSs, its changes after cisplatin-based chemotherapy, and its associations with adverse health outcomes. A major strength of our investigation is that it was conducted within the Platinum Study, a rigorous multicenter investigation of TCSs with a high patient participation rate (93%), detailed medical record abstraction, physical examinations, and an assessment of adverse health outcomes and health behaviors using validated questionnaires (5,13). Another strength is that fat compartments were quantified on CT and that the applied method was previously validated in the Framingham Study (16); it provides a considerably more accurate representation of body fat distribution than clinical surrogates such as tape-measured waist and hip circumferences (35).

The major limitation of our study is its retrospective design, with 455 (71%) of 639 MSKCC patients in the Platinum Study having an eligible prechemotherapy CT scan for the current investigation. It is unlikely, however, that selection bias was introduced, because the acquisition of pretreatment CTs is typically not related to the later development of CMD. Similarly, quantitative examination of changes in body fat distribution after chemotherapy necessitated limitation to those with an eligible follow-up CT. Because CT scan intervals are more frequent in the early period postchemotherapy, these patients had a shorter interval between chemotherapy and questionnaire completion. However, importantly, a systematic examination of other variables (eg, baseline age, BMI, fat distribution) (Supplementary Table 1, available online) found no statistically significant differences between the follow-up CT subgroup and the remainder of the cohort. In addition, despite this short follow-up interval, there was still a statistically significant increase in BMI following chemotherapy, a finding supported by clinical experience.

Another limitation is that fat distribution was quantified on a single transverse CT slice as validated in a previous prospective study (16); although this surrogate might not perfectly represent body fat volumes, the 3-dimensional measurements of fat compartments on CT scans are considerably more labor intensive and would be difficult to integrate into a clinical workflow. Thus, the applied measurement method represents a reasonable trade-off between accuracy and practicability. Among patients with very little fat, the calculation of a VAT-to-SAT ratio might be susceptible to even small measurement variabilities, as reflected by the presence of outliers in normal or underweight patients. Although CT-derived quantifiers of body fat distribution must therefore be carefully interpreted in normal-weight or underweight individuals, our data also suggest that this is of low clinical significance given the low cardiometabolic risk of this group. Additionally, because the vast majority of our patients received 4 cycles of chemotherapy, we were not able to assess the potential confounding effect of the number of chemotherapy cycles on CMD risk (36). Other factors that potentially confound the reported associations of body fat distribution and CMD risk in TCSs but were also not included in the current analysis include testosterone levels and hypogonadism (37,38) as well as the effects of physical exercise (39,40). Finally, due to self-reported medication use as criterion for CMD and because CMD might be underdiagnosed in young individuals (41-43), the incidence of CMD might be underestimated in our patients.

Central adiposity before cisplatin-based chemotherapy is a cardiometabolic risk factor for TCSs. The cardiometabolic health effects of an unfavorable body fat distribution with preferential fat accumulation in the visceral compartment are most pronounced in young or obese individuals. Importantly, these findings suggest that young or obese germ cell tumor patients with high VAT-to-SAT ratios before chemotherapy may benefit most from targeted CMD reduction interventions (blood pressure and lipid control, diet, and exercise). Future studies with long-term follow-up should evaluate the importance of dynamic changes in fat distribution at various time points postchemotherapy and test interventional strategies in this population.

## Funding

This work was supported by National Institute of Health/National Cancer Institute (P30 CA008748 to MSKCC, R01 CA 157823 to LBT and HDS).

## Notes

**Role of the funder:** The funders had no role in the design of the study; the collection, analysis, and interpretation of the data; the writing of the manuscript; and the decision to submit the manuscript for publication.

**Disclosures:** The authors have no conflict of interest to declare.

**Author contributions:** Conceptualization: A.G.W., D.R.F., H.A.V. Data Curation: A.G.W, P.C.D., J.Z., M.C., M.B. Formal Analysis: A.G.W., J.Z., M.C., D.R.F., H.A.V. Funding Acquisition: D.R.F., H.A.V., L.B.T., H.D.S. Investigation: A.G.W., P.C.D., L.B.T., M.B., D.R.F., H.A.V. Methodology: A.G.W., J.Z., M.C., D.R.F., C.C. Project Administration: P.C.D., L.B.T., H.D.S., D.R.F., H.A.V. Resources: P.C.D., L.B.T., D.R.F., H.A.V. Supervision: L.B.T., C.C., D.R.F., H.A.V. Writing – original draft: A.G.W., J.Z., M.C., D.R.F., H.A.V. Writing – review and editing: all authors.

**Prior presentations:** This work was presented as an abstract during a Poster Discussion Session of the 2021 American Society of Clinical Oncology (ASCO) Annual Meeting (Abstract 5019).

## Data Availability

Data contains protected health information and are therefore not available.

## Supplementary Material

pkac030_Supplementary_Data
