# Supplementary material for: Associations of Body Fat Distribution and Cardiometabolic Risk of Testicular Cancer Survivors After Cisplatin-Based Chemotherapy
Source: JNCI Cancer Spectr. 2022 May 13;6(4):pkac030. doi: 10.1093/jncics/pkac030 (PMC9263534; doi:10.1093/jncics/pkac030)
Supplement: pkac030_Supplementary_Data [file pkac030_supplementary_data.pdf]

SUPPLEMENTARY MATERIAL

Supplementary Table 1. Comparison of patients with and without a follow-up CT within 8 weeks of the Platinum Study questionnaire.

Variable	Follow-up CT available (n=108)	No follow-up CT (n=347)	*P*
Baseline pre-chemotherapy characteristics			
Median age (IQR), years	34 (27, 41)	31 (26, 39)	0.10[a]
Median BMI (IQR), kg/m^2	26 (24, 29)	26 (24, 29)	0.91[a]
Median Systolic blood pressure (IQR), mmHg	123 (117, 132)	121 (113, 130)	0.07[a]
Median Visceral Adipose Tissue area (IQR), cm^2	75 (42, 145)	76 (36, 138)	0.68[a]
Median Subcutaneous Adipose Tissue area (IQR), cm^2	157 (102, 218)	155 (98, 233)	0.84[a]
VAT/SAT ratio	0.53 (0.34, 0.78)	0.48 (0.30, 0.74)	0.35[a]
Cardiometabolic disease, No. (%)	12 (11.1)	26 (7.5)	0.32[b]
Median Interval between Chemotherapy and study questionnaire (IQR), months	18 (15, 31)	36 (17, 66)	<0.001[a]
Median change in BMI between chemotherapy and study questionnaire (IQR), kg/m^2	+0.71 (-0.40, +2.30)	+0.95 (-0.09, +2.26)	0.30[a]
Median Framingham Risk[c] at questionnaire (IQR), %	3.6 (1.5, 7.4)	3.3 (1.7, 6.8)	0.86[a]

[a]*P* values are derived from Wilcoxon two-sample tests. All tests were 2-sided. BMI= Body Mass Index; CT= computed tomography; SAT= subcutaneous adipose tissue; VAT= visceral adipose tissue.

[b] *P* values are derived from two-sided Chi-squared test.

[c] Framingham Heart Study estimated 10-year risk for atherosclerotic cardiovascular disease (office- based calculator)
